# Insulin Resistance, Metabolic Syndrome, and Inflammatory Skin Disease

**DOI:** 10.3390/jcm15010330

**Published:** 2026-01-01

**Authors:** Krisha Tripathy, Ajay S. Dulai, Mildred Min, Raja K. Sivamani

**Affiliations:** 1 Vagelos College of Physicians and Surgeons, Columbia University, New York, NY 10027, USA; 2 Integrative Skin Science and Research, Sacramento, CA 95815, USA; 3 Integrative Research Institute, Sacramento, CA 95819, USA; 4 College of Medicine, California Northstate University, Elk Grove, CA 95757, USA; 5 Pacific Skin Institute, Sacramento, CA 95815, USA; 6 Department of Dermatology, University of California-Davis, Sacramento, CA 95816, USA

**Keywords:** glucose control, insulin resistance, metabolic syndrome, diabetes, psoriasis, acne, hidradenitis suppurativa, acanthosis nigricans, seborrheic dermatitis

## Abstract

**Background/Objectives**: The skin is an important indicator of overall health, and its relationship with insulin resistance (IR) and metabolic syndrome (MetS) has garnered increasing attention. This review explores the connection between glucose dysregulation and various dermatological conditions, aiming to highlight integrative approaches for management. **Methods**: A comprehensive literature search was conducted in June and July 2024 across PubMed, Google Scholar, and Embase. Peer-reviewed studies on glucose dysregulation in dermatology were identified using terms such as “insulin,” “metabolic syndrome,” and “dermatological manifestations.” Relevant studies were selected based on their contributions to understanding these relationships. **Results**: The review identified significant associations between glucose dysregulation, MetS, and conditions such as psoriasis, acne, acanthosis nigricans, seborrheic dermatitis, and hidradenitis suppurativa. Key findings indicated that elevated insulin levels and inflammatory markers correlate with the severity of these skin disorders. Notably, dietary interventions and probiotics show potential in modulating inflammation and improving metabolic health. **Conclusions**: There is a clear link between glucose dysregulation and several dermatological conditions, underscoring the importance of a holistic treatment approach. By addressing glucose control and incorporating lifestyle modifications, clinicians can improve patient outcomes and mitigate the complications associated with IR and MetS. Further research is essential to refine these integrative strategies and assess their effectiveness in clinical practice.

## 1. Introduction

As the body’s primary barrier, skin is highly susceptible to environmental changes. Beyond this, the skin can also reflect internal and systemic health. Metabolic syndrome (MetS), which is a condition that encompasses factors including abdominal obesity, IR, dyslipidemia, and high blood pressure [1], has been posited to significantly contribute to the development of various dermatoses. A systematic review evaluated the association between MetS and common skin disorders with thirty-seven studies involving 13,830 participants [2]. They found a higher prevalence of MetS in patients with psoriasis (PsO), hidradenitis suppurativa (HS), vitiligo, androgenetic alopecia, and lichen planus LP. Additionally, individuals with seborrheic dermatitis and rosacea demonstrated increased IR, high blood pressure, and elevated blood lipids. Meta-analysis revealed a significant link between MetS and skin diseases (pooled odds ratio [OR]: 3.28, 95% confidence interval: 2.62–4.10) [2]. These findings highlight the need for comprehensive management by dermatologists and multidisciplinary teams to prevent severe complications associated with MetS across different dermatologic conditions (Table 1). In this review, we fill gaps in existing literature by emphasizing the role of integrative therapies in the associations between metabolic dysregulation and various dermatoses, offering a holistic approach that complements previous systematic reviews. Integrative medicine is an approach that combines conventional medical practices with complementary therapies to promote holistic health and wellness, particularly in managing conditions such as those related to glucose control in dermatology. In this integrative approach we emphasize all aspects of health that contribute to inflammatory skin diseases and all factors contributing to glucose regulation including hormones, stress, the gut microbiome, diet, and lifestyle factors.

## 2. Materials and Methods

We searched PubMed, Google Scholar, and Embase databases for peer-reviewed clinical studies and reviews of glucose dysregulation in dermatologic manifestations. Search terms such as “insulin,” “dermatology,” “integrative medicine,” “gut microbiome,” “psoriasis,” “diabetes,” “glucose control,” and “metabolic syndrome” were included in the search strategy. Studies pertaining to the body’s regulation of glucose, disruptions in glucose control, dermatologic manifestations of glucose dysregulation, and related integrative approaches were included. Exclusion criteria included studies not published in peer-reviewed journals, those with inadequate methodological rigor, and research focusing on populations outside the scope of dermatological conditions related to glucose control. Reference sections were additionally searched for further related studies that fell within inclusion criteria.

## 3. Results and Discussion

### 3.1. Psoriasis

Psoriasis (PsO) is a common chronic recurrent immune-mediated skin disease that is linked to systemic inflammation. PsO is often linked to cardio-metabolic complications, but has gained more attention for its link to IR due to the widespread inflammation involved in its pathogenesis [18].

A study of 425 individuals: 86 with psoriasis, 69 with both PsO and type 2 diabetes mellitus (T2DM), 120 with T2DM, and 150 healthy participants aimed to analyze IR, lipid irregularities, and cardiovascular risk indicators in patients with PsO, both with and without T2DM [3]. Key metrics included the Psoriasis Area and Severity Index (PASI), body mass index (BMI), IR parameters (HbA1c, fasting plasma glucose (FPG), fasting plasma insulin (FPI), and HOMA index), lipid profiles, and more. Findings indicated that FPG, HbA1c, and HOMA-IR levels were significantly higher in individuals with both PsO and T2DM compared to those with only PsO [3]. FPI levels were elevated in psoriatic diabetics relative to psoriatics, and the FPI was higher in psoriatics than the control group [3]. Although the study faced limitations of age differences between control and study groups, it emphasized a link between PsO and T2DM, suggesting that IR is a key factor in PsO comorbidities.

Another study examined serum and tissue levels of interleukin IL-9 and its receptor in PsO patients with MetS to analyze how IL-9 levels correlate with the severity of PsO. FPG, insulin, lipid profiles, and serum levels of IL-9 and IL-17A were measured in PsO patients with and without MetS, and results showed that serum and tissue levels of IL-9 and its receptor were elevated (*p* < 0.05) in PsO patients with MetS. IL-9 levels were also positively associated with waist circumference (WC) (*p* < 0.001), BMI (*p* = 0.002), IR (*p* = 0.036), blood pressure (*p* < 0.001), and triglycerides (*p* = 0.001), and were negatively associated with high-density lipoprotein cholesterol levels (*p* = 0.001) [4]. These findings suggest that elevated IL-9 levels may play a role in correlating PsO and MetS.

One study assessed the impact of administering *Bifidobacterium infantis* 35624 orally for 6–8 weeks across patients with ulcerative colitis (UC), chronic fatigue syndrome (CFS), and PsO in separate randomized, double-blind, placebo-controlled trials [5]. Its effects on immunological biomarkers were studied in healthy subjects. Initially, all patient groups exhibited elevated levels of inflammatory markers such as tumor necrosis factor α (TNF-α), C-reactive protein (CRP), and interleukin-6 (IL-6) compared to healthy controls [5]. *B. infantis* 35624 supplementation significantly reduced plasma CRP levels in all three conditions, decreased TNF-α levels in CFS and PsO groups, and decreased IL-6 in UC and CFS [5]. Moreover, in healthy subjects, *B. infantis* 35624 led to a notable reduction in TNF-α and IL-6 secretion by peripheral blood mononuclear cells stimulated with lipopolysaccharide [5]. Although these findings underscore the microbe’s ability to modulate systemic inflammation in diverse disorders, higher-quality evidence from dermatology-specific studies is needed to understand direct implications on inflammatory dermatoses.

Interestingly, the literature points to intermittent fasting as a practice to improve PsO and PsO-related pathologies. One systematic review consisting of 45 studies included a study trending circulating pro-inflammatory cytokines during Ramadan fasting and found that certain pro-inflammatory cytokines (IL-1b, IL-6, and TNF-a) significantly decreased while healthy volunteers fasted (n = 50) [6]. Specifically, looking at PsO, a multicenter study evaluated the impact of intermittent fasting on psoriatic arthritis, enthesitis, and dactylitis, and found a decrease in CRP levels, along with improvements in enthesitis and dactylitis using Leeds Enthesitis Index (LEI) and dactylitis severity score (DSS), respectively [7]. The correlation between intermittent fasting and decreased inflammatory markers associated with PsO warrants further investigation to better understand the role of diet modification and inflammatory dermatoses.

### 3.2. Acne

Acne is a chronic inflammatory disorder affecting the pilosebaceous unit [8]. The relationship between acne, MetS, and IR is one that has been explored in the current literature. Acne can serve as a marker for the effects of a Western diet high in insulin-stimulating foods. Diets with a high glycemic load increase blood glucose level, prompting the pancreas to release more insulin [9]. This heightened insulin level boosts the production of insulin-like growth factors (IGF), leading to increased fibroblast and keratinocyte proliferation, comedone formation, androgen levels, and inflammation [9]. Clinical studies have further clarified the relationship between insulin sensitivity and acne. A study involving 162 acne patients over 20 years old and 78 healthy, age- and sex-matched controls measured parameters such as WC, BMI, blood pressure, fasting glucose and insulin, HDL, LDL, triglycerides, and total cholesterol [8]. The findings showed that acne patients had a significantly higher prevalence of MetS (12.3% vs. 2.6%, *p* = 0.014) and IR (11.7% vs. 3.8%, *p* = 0.047) compared to controls, with both conditions being more common in those with severe acne [8]. WC was also identified as an independent risk factor for both MetS and IR in patients with acne [8]. This suggests that severe adult acne is associated with metabolic markers of MetS, IR, and that dermatologists should consider this when taking care of these patient populations.

A meta-analysis indicated that dairy intake, especially milk, is linked to a higher risk of acne, likely due to the insulinotropic effects of whey proteins and IGF-1 stimulation by casein [10]. Unlike milk, fermented dairy products like yogurt do not significantly impact acne because fermentation reduces milk-mediated mTORC1 signaling [10]. On the other hand, a diet rich in fish, fruits, and vegetables may reduce acne risk due to the omega-3 fatty acids and high fiber content, which lower IGF-1 levels [10]. These findings suggest that further research is needed to explore the specific impacts of dairy intake on glucose tolerance and insulin sensitivity in relation to inflammatory dermatoses. Another study evaluated metformin’s role in treating AV by reducing insulin-like growth factor 1 (IGF-1) levels in 50 patients aged 16 to 30 years. After four months of metformin therapy, patients showed clinical improvement in the global acne grading system (GAGS) scores and a significant decrease in IGF-1 levels [11]. This suggests that IGF-1 may play a role in acne pathogenesis and that metformin may be an effective treatment option for dermatologists to consider.

### 3.3. Acanthosis Nigricans

Acanthosis nigricans manifests as velvety, darkened patches of skin, commonly found in intertriginous regions. This condition often presents with indistinct borders and usually appears in skin folds such as the back of the neck, armpits, and groin, often accompanied by skin thickening [12]. For example, facial acanthosis nigricans (FAN), also known as metabolic melanosis or metabolic melasma, presents as brown to black pigmentation with blurred, ill-defined borders typically on the forehead, temples, and cheeks [13].

Over the past decade, studies have increasingly linked FAN to IR and obesity, making it a visible marker of MetS. The condition arises from the proliferation of epidermal keratinocytes and dermal fibroblasts, driven by high insulin levels that activate insulin-like growth factor 1 receptors (IGF-1Rs) [13]. This leads to the characteristic skin changes seen in acanthosis nigricans. Additionally, hyperinsulinemia elevates free IGF-1 levels in the blood by reducing insulin-like growth binding proteins (IGFBPs), further promoting FAN development. Other mediators such as pigment epithelium derived factor (PEDF) are linked to IR through its stimulation of adipocyte lipolysis and activating proinflammatory kinases, with a noted correlation between FPI, HOMA-IR, and PEDF levels [14]. PEDF is abundant in facial melanosomes, contributing to increased pigmentation, which can decrease with weight loss and lifestyle changes [14]. This data suggests that targeting glucose regulation and insulin sensitivity may be options for managing this acanthosis nigricans.

### 3.4. Seborrheic Dermatitis

Seborrheic dermatitis is a chronic and relapsing inflammatory skin disease identified with scaling, erythematous plaques. Understanding of the systemic effects of seborrheic dermatitis (SD) as a chronic inflammatory skin disease remains limited, but some studies have attempted to explain it.

One study of 53 patients over the age of 18 diagnosed with SD and 50 healthy controls aimed to evaluate MetS and glucose metabolism disorders in SD patients [15]. Results showed significantly higher weight, WC, family history of SD and metabolic disorders in the SD group compared to the control group. FPI, triglyceride levels, HOMA-IR, and OGTT 2-h PG were also significantly higher in SD patients. Moreover, IR was significantly more common in the SD group (58.49%) compared to controls (22%) [15]. These findings support the idea that, possibly due to the common inflammatory pathways, there is a link between SD and IR. A different meta-analysis of 37 studies involving 13,830 participants also aimed to evaluate the link between MetS and SD and other skin conditions [2]. Findings indicated that individuals with SD, along with rosacea, exhibit a higher propensity for IR, hypertension, and elevated blood lipid levels [2]. The meta-analysis demonstrated a significant association between MetS and skin diseases, with a pooled odds ratio (OR) of 3.28. Specifically, SD had an OR of 2.45, suggesting a notable correlation between SD and MetS [2]. Thus, taking a more holistic approach to treating SD patients may prove fruitful.

### 3.5. Hidradenitis Suppurativa

HS is another chronic inflammatory skin condition marked by painful lesions in areas rich in apocrine glands, such as the inguinal, axillary, sub-mammary, and anogenital regions [16]. HS frequently occurs alongside obesity, MetS, T2DM, IR, and polycystic ovarian syndrome. Many patients often seek complementary non-medical interventions, particularly dietary changes, alongside conventional treatments due to the chronicity of the disease [16].

The “skin-gut axis” suggests a connection between the gastrointestinal microbiome and skin health, with diet influencing insulin and IGF-1 pathways that may affect HS pathogenesis. Importantly, beyond microbial or metabolic signaling alone, the skin-gut connection is also mediated by a shared neuroimmune regulatory network with developmental origins in the neural ectoderm [19]. Neural crest-derived cells—including cutaneous sensory neurons, enteric neurons, and associated immune-modulating cells—form a persistent bidirectional communication system that links gut-derived inflammatory or metabolic cues to cutaneous immune activity [19]. One review suggests that adopting a Mediterranean lifestyle and diet alongside traditional therapies may offer cost-effective benefits [17]. Conversely, high glycemic index foods and dairy could exacerbate HS symptoms, possibly through mechanisms involving IR and inflammation [17]. Zinc, brewer’s yeast-free diet, B12 supplementation, intermittent fasting, and refined sugar and dairy intake were all concluded to be points of future interest in this study [17]. Ultimately, the study emphasized the limited evidence supporting a significant role of diet alone in managing HS, and highlight the necessity of additional medical therapy for effective treatment [17]. Metformin also reduces inflammatory cytokines such as TNF-α and IL-17 which are involved in HS [20]. A systematic review of 133 HS patients across six studies, predominantly female and overweight or obese, showed that metformin monotherapy led to improvement in most cases, with only mild and transient side effects [20]. Given its efficacy and tolerability, further clinical trials comparing metformin with placebo for HS treatment are warranted.

### 3.6. Androgenic Alopecia

Androgenetic alopecia (AGA) is a chronic, androgen-dependent miniaturization of scalp hair follicles driven by dihydrotestosterone (DHT) sensitivity, with growing evidence implicating MetS and IR as systemic contributors. A systematic review and meta-analysis of 19 case–control studies (2531 participants) evaluated the association between androgenetic alopecia (AGA) and MetS and compared metabolic profiles with healthy controls [21]. Studies from nine countries used standardized MetS definitions, assessing BMI, WC, FPG, HDL-C, triglycerides, and blood pressure. Pooled analysis showed AGA was linked to higher MetS prevalence (OR 3.46, 95% CI 2.38–5.05; *p* < 0.001), with female sex, early-onset AGA (<36 years), and African ethnicity conferring even greater risk [21]. AGA patients had higher BMI, WC, triglycerides, blood pressure, fasting glucose, and lower HDL compared with controls. Heterogeneity was moderate (I^2^ = 63.7%), and meta-regression found no effect of publication year, region, study quality, or sample size [21]. Limitations included observational designs, variable alopecia grading, and demographic differences. These results support AGA as a clinical marker of metabolic dysfunction, emphasizing the need for metabolic screening and further large-scale prospective studies.

A large practice-based case–control study of early-onset AGA in Finnish men (n = 154 cases; n = 154 age-matched controls) demonstrated clear co-aggregation of IR-related conditions [22]. AGA patients had higher BMI (27.1 vs. 25.1 kg/m^2^; *p* < 0.0001) and were significantly more likely to be overweight (OR 2.90) or obese (OR 2.46). Cardiometabolic medication use was also elevated, including antihypertensives (OR 2.09) and lipid-lowering therapy (OR 4.45). Clustered IR risk factors—defined as ≥3 abnormalities (dyslipidemia, abnormal glucose metabolism, BMI ≥ 30, or SBP ≥ 160 mmHg)—were markedly more common in AGA (13.2% vs. 3.1%; OR 4.79), and hyperinsulinemia was increased (44.8% vs. 29.8%; OR 1.91) [22]. A complementary case–control study of 100 men with Hamilton–Norwood stage III–VII AGA found significantly higher fasting insulin, HOMA-IR, triglycerides, waist circumference, and blood pressure compared with matched controls, with strong associations between AGA, IR (*p* < 0.001), and full MetS (*p* = 0.002) [23]. Logistic regression identified waist circumference > 102 cm as the most powerful predictor of MetS, emphasizing the contribution of central adiposity to the dermatologic presentation.

Mechanistically, insulin signaling provides a plausible biological link between IR and androgen-driven follicular miniaturization. Hyperinsulinemia suppresses sex hormone-binding globulin, thereby increasing circulating free testosterone and enhancing androgen action at the follicle [24,25,26]. Insulin also modulates steroidogenesis within Leydig cells, influencing genes involved in androgen synthesis and leading to the secretion of testosterone and DHT [27,28,29,30]. This convergence of elevated bioavailable androgens, metabolic dysfunction, and vascular/inflammatory changes supports the model of AGA as a cutaneous marker of underlying IR.

Correcting insulin resistance has been associated with improvement in androgenic alopecia-related hair loss. A recent case report described a 57-year-old man with clinically and trichoscopically confirmed AGA and biochemical insulin resistance who demonstrated marked improvement in hair density within 6 months of initiating tirzepatide monotherapy, with continued regrowth over 1 year alongside normalization of insulin resistance and significant weight loss [31]. This temporal improvement supports a potential mechanistic link between hyperinsulinemia and follicular miniaturization, as insulin resistance amplifies androgen signaling, impair microvascular perfusion, and promote dihydrotestosterone-mediated follicular apoptosis [32]. Although prior studies have been largely correlative, this report provides early clinical evidence that improving insulin sensitivity, particularly with GLP-1/GIP receptor agonists, may mitigate pathogenic pathways contributing to AGA [31]. Further prospective studies are warranted to clarify causality and define the role of metabolic-targeted therapies in hair loss management.

### 3.7. Atopic Dermatitis

Atopic dermatitis (AD) is a chronic, immune-mediated inflammatory skin disease characterized by epidermal barrier dysfunction, type 2-skewed cytokine activity, and recurrent flares [33]. AD’s systemic inflammatory profile and alterations in lipid homeostasis have raised interest in potential links to MetS, obesity, and IR, particularly in individuals with more severe or persistent disease.

A controlled physiologic case–control study (n = 32) compared 16 non-obese, non-diabetic adults with long-standing mild–moderate AD (EASI 8.5 ± 1.0; disease duration 28 ± 3 yrs) to 16 age-, gender-, and BMI-matched controls. Participants underwent a 3-h hyperinsulinemic–euglycemic clamp (insulin infusion 40 mU/m^2^/min) and a 3-h 75 g OGTT with serial measurements of glucose, insulin, C-peptide, glucagon, GIP, and GLP-1. Fasting glucose (5.1 ± 0.1 vs. 5.0 ± 0.1 mmol/L), HbA1c (5.0 ± 0.1% vs. 4.9 ± 0.1%), and all OGTT-derived total AUCs were comparable between groups, with no differences in insulin secretion rate, β-cell function (insulinogenic index 235 vs. 167 pmol/mmol), or glucagon/GIP responses. The only hormonal divergence was higher GLP-1 in AD (tAUC 5039 ± 204 vs. 4333 ± 119 pmol·min/L; *p* = 0.006) [34]. Clamp-derived insulin sensitivity did not differ (M-value 9.2 ± 0.6 vs. 9.8 ± 0.8 mg/kg/min; *p* = 0.541, 95% CI −1.51 to 2.60). Overall, adults with mild–moderate AD showed no impairment in insulin sensitivity or glucose homeostasis [34]. In contrast, a cross-sectional cohort (n = 233,628) demonstrated that while AD overall was associated with higher dyslipidemia but lower diabetes and MetS, adults with moderate-to-severe AD exhibited substantially higher prevalence of MetS (17% vs. 9.4%), obesity (22.2% vs. 18.6%, *p* < 0.001), diabetes (15.9% vs. 9.2%, *p* < 0.001), hypertension (27.9% vs. 15.3%, *p* < 0.001), and dyslipidemia (47.1% vs. 28.5%, *p* < 0.001), with multivariate analysis confirming an independent association (*p* = 0.04) [35]. A third nationwide study in Korean adults (n = 5007) found that women with AD had significantly higher odds of MetS (OR 2.92), central obesity (OR 1.73), and hypertriglyceridemia (OR 2.20), indicating possible sex-specific metabolic vulnerability [36]. Collectively, these findings suggest that metabolic risk in AD is severity- and population-dependent, with severe and female-predominant cohorts showing the strongest associations.

Biologically, several mechanisms may link AD to metabolic dysregulation. Systemic type 2 inflammation can alter lipid profiles, reduce adipocyte insulin sensitivity, and influence hepatic glucose handling, while chronic barrier dysfunction may modify cytokine signaling, oxidative stress, and neuroimmune pathways implicated in MetS. Glucose itself exerts osmotic effects relevant to AD: elevations in extracellular glucose increase tissue tonicity, drawing water from keratinocytes [37,38]; GLUT- and SGLT-mediated glucose transport generates diffusion-osmotic and convective water shifts [39]; and studies demonstrate that sweat glucose is elevated during acute AD, delaying transepidermal water loss recovery after barrier injury [40]. These osmotic processes are not central drivers of AD but may worsen xerosis and prolong flares under metabolic stress.

Although robust data on the impact of glucose levels on AD are lacking, patient-reported data suggest that high-sugar diets can trigger or worsen symptoms. In a cross-sectional survey of 169 AD patients using a 61-item questionnaire adapted from the National Health and Nutrition Examination Survey, 87% reported trying dietary exclusions—most commonly junk foods (68%), dairy (49.7%), and gluten (49%)—with the greatest perceived improvements seen after removing white flour products (53.6%) and gluten (51.4%) [41]. When assessing perceived dietary triggers and dietary modifications, sugar was identified as a trigger by 16.5% of respondents—making it the fourth most frequently reported trigger, following dairy (24.8%), gluten (18.3%), and alcohol (17.1%) [41]. Another pilot study specifically assessed for differences in glucose levels and insulin levels among 9 and 15 year old with and without AD (PMID 38371671). The authors noted that while there were no significant changes noted in glucose levels in either group, insulin levels were higher in those with moderate/severe AD compared to those without AD in the 15 year-old group. Future studies should focus on the role of insulin and insulin resistance as an important comparator in addition to assessing the role of glucose in AD.

### 3.8. Candida Balanitis

Candida balanitis is an inflammatory infection of the glans penis characterized by erythema, pruritus, discharge, and epithelial irritation, most commonly caused by Candida albicans [42]. It is strongly linked to systemic metabolic disturbances—particularly diabetes, hyperglycemia, and glucosuria—in which impaired innate immunity, elevated local glucose availability, and altered mucocutaneous barrier function create a permissive environment for fungal overgrowth.

A large population-based cohort examined 271,840 adults (146,603 men; 73,383 with T2DM) to determine 1-year incidence rates of genital Candida infections [43]. Among men with T2DM, balanitis incidence was 8.4 per 1000 person-years (95% CI 7.8–9.1), compared with 2.5/1000 PY in matched non-diabetic controls. This corresponded to an adjusted RR of 2.85 (95% CI 2.39–3.39), with the strongest relative risk observed in younger adults [43]. Glycemic control strongly influenced risk: infection rates increased stepwise across HbA1c strata, and men with poor control (HbA1c > 8%) had substantially higher incidence than those with fair control [43]. In another cross-sectional study of 478 men attending an STD clinic, the prevalence of candida colonization was 26.2% and candida balanitis was 18%, with 45.5% of men with balanitis showing a positive Candida culture [42]. T2DM was significantly more common in men with candida balanitis than in asymptomatic men (13.8% vs. 4.2%, *p* < 0.001) and was an independent risk factor for symptomatic infection (multivariate OR = 19.39; 95% CI: 7.79–48.27). Other risk factors included age over 40 years and isolation of >10 Candida colonies (OR = 9.59; 95% CI: 2.68–34.26), whereas sexual behavior, STI status, and impaired glucose tolerance were not significantly associated [42]. These findings highlight the strong link between T2DM and the development of candida balanitis.

Mechanistically, elevated circulating and local glucose levels directly promote Candida proliferation, adhesion, and tissue persistence. Experimental models demonstrated that adding high concentrations of glucose (up to 3000 mg/dL) to urine increased viable Candida counts more than ten-fold, while concurrently raising minimum inhibitory concentrations of azoles and 5-fluorocytosine [44]. This was accompanied by upregulation of antifungal resistance genes (ERG11, CDR1, CDR2, MDR1), suggesting that hyperglycosuria not only fuels growth but also induces adaptive resistance pathways [44]. In parallel, flow-cytometry studies confirmed a dose-dependent enhancement of C. albicans growth in high-glucose environments, while fructose inhibited growth, raising intriguing nutritional considerations [45].

Although direct studies on dietary intervention for Candida balanitis are lacking, restricting refined sugars has shown benefit in related conditions such as recurrent vulvovaginal candidiasis [46]. Additionally, animal models suggest that a ketogenic (low-carbohydrate) diet can enhance the efficacy of antifungal therapy against Candida infections. In murine models of Cryptococcus neoformans and Candida albicans infection, a ketogenic diet combined with fluconazole significantly reduced fungal burden compared to fluconazole on a conventional diet: brain (2.66 ± 0.289 log_10_ reduction) and lung (1.72 ± 0.399) for *C. neoformans*, and kidney (2.37 ± 0.770) for C. albicans [47]. The ketogenic diet also increased fluconazole concentrations in plasma and brain, enhancing its efficacy at lower doses. These findings suggest that a high-fat, low-carbohydrate ketogenic diet can potentiate fluconazole treatment and may represent a novel strategy to improve antifungal therapy.

Ultimately, across metabolic, microbial, and pharmacologic domains, hyperglycemia emerges as a central driver of Candida balanitis risk, colonization density, and treatment resistance, making glycemic optimization a key component of prevention and therapy.

### 3.9. Limitations

Limitations of the study include variability in how insulin resistance, metabolic syndrome, and dermatologic disease severity were defined and measured across studies. Evidence supporting integrative approaches—such as dietary modification, probiotics, and intermittent fasting—largely derives from early-phase or heterogeneous studies, which limits the strength of conclusions. Additionally, publication bias and the underrepresentation of diverse populations may affect the generalizability of the findings. These limitations outline the current boundaries of the evidence base and indicate areas where more standardized, rigorous, and inclusive research is needed.

## 4. Conclusions

Glucose regulation is fundamental to skin health, minimizing skin complications, improving wound healing, and alleviating the symptoms of inflammatory dermatoses. The integration of glucose control strategies into dermatologic care represents an important paradigm shift towards comprehensive patient management. The evidence presented indicates that elevated insulin levels and inflammatory markers significantly correlate with the severity of these skin conditions, suggesting a bidirectional link that warrants attention in clinical practice. By recognizing the interplay between metabolic health and dermatologic conditions such as acne, PsO, and HS, clinicians can adopt a proactive approach that targets underlying metabolic dysregulations.

Integrative approaches, including dietary modifications and the use of probiotics, have shown promise in managing both metabolic health and dermatological symptoms. By adopting a holistic treatment framework that addresses glucose regulation and insulin sensitivity, dermatologists can enhance patient outcomes and potentially prevent the progression of related comorbidities. Intermittent fasting, dairy intake, sleep hygiene, and weight and aerobic training have been implicated in improving glucose control and may be explored during shared decision-making discussions between patients and their physicians.

Moving forward, continued research and clinical innovation will help optimize these approaches and provide clarification on optimal care for patients experiencing diverse skin manifestations of metabolic dysregulation.

## Figures and Tables

**Table 1:** Association between chronic inflammatory skin conditions and insulin resistance/metabolic syndrome.

Condition	Key Findings	Associated Biomarkers/Factors	Links to Insulin Resistance/Metabolic Syndrome
**Psoriasis (PsO)** [3,4,5,6,7]	Elevated IR (IR—Insulin Resistance) markers (HbA1c (FPG—Fasting Plasma Glucose), FPG (FPG—Fasting Plasma Glucose), HOMA-IR (HOMA-IR—Homeostatic Model Assessment of Insulin Resistance)) in PsO patients with T2DM (T2DM—Type 2 Diabetes Mellitus); elevated IL-9 (IL-9—Interleukin 9) in PsO patients with MetS (MetS—Metabolic Syndrome) linked to severity of PsO and IR.	PASI (PASI—Psoriasis Area and Severity Index), FPG, HbA1c, FPI (FPI—Fasting Plasma Insulin), HOMA-IR, IL-9, IL-17 (IL-17—Interleukin 17)	PsO is associated with markers of IR that may precede T2DM, with IL-9 and systemic inflammation playing a role.
**Acne** [8,9,10,11]	Higher prevalence of MetS and IR in severe acne patients; dietary factors such as dairy intake may worsen acne due to insulinotropic effects.	WC (WC—Waist Circumference), BMI (BMI—Body Mass Index), FPG, FPI, HDL (HDL—High-Density Lipoprotein), LDL (LDL—Low-Density Lipoprotein), triglycerides	Severe acne has correlation with MetS and IR; dietary factors like dairy and glycemic load influence insulin sensitivity.
**Acanthosis Nigricans (AN)** [12,13,14]	Elevated FPI and HOMA-IR levels associated with FAN (FAN—Facial Acanthosis Nigricans); hyperinsulinemia promotes epidermal keratinocyte proliferation.	FPI, HOMA-IR, PEDF (PEDF—Pigment Epithelium-Derived Factor)	FAN is associated with markers of IR and obesity, with high insulin levels contributing to the skin changes seen in AN.
**Seborrheic Dermatitis (SD)** [2,15]	Higher WC, FPI, triglycerides, and HOMA-IR in SD patients compared to controls; MetS more common in SD patients.	WC, FPI, HOMA-IR, triglycerides, OGTT (OGTT—Oral Glucose Tolerance Test)	SD is linked to markers of IR and MetS, highlighting the need for a holistic approach to treatment.
**Hidradenitis Suppurativa (HS)** [16,17]	HS associated with obesity, MetS, T2DM, and IR; diet and lifestyle factors, including Mediterranean diet, may modulate IR and inflammation.	FPI, TNF-α (TNF-α—Tumor Necrosis Factor Alpha), IL-17	HS patients show significant comorbidities with IR and MetS; metformin and dietary interventions offer potential treatments.
**Androgenic Alopecia (AA)**	Higher prevalence of MetS in AGA; patients show elevated BMI, WC, triglycerides, blood pressure, fasting glucose, and lower HDL.	BMI, WC, FPG, FPI, HOMA-IR, HDL, triglycerides, blood pressure	AGA is associated with markers of underlying IR; hyperinsulinemia increases free androgen levels and contributes to follicular miniaturization.
**Atopic** **Dermatitis (AD)**	Moderate-to-severe AD associated with higher prevalence of MetS, obesity, diabetes, hypertension, and dyslipidemia; insulin sensitivity preserved in mild AD.	BMI, WC, FPG, insulin, HbA1c, triglycerides, HDL, GLP-1 (GLP-1—Glucagon-Like Peptide 1)	Moderate-to-severe AD, particularly in females, is linked to markers of IR and MetS; systemic inflammation and barrier dysfunction may exacerbate metabolic dysregulation.
**Candida** **Balanitis**	Incidence of balanitis higher in men with T2DM; risk rises with poor glycemic control.	HbA1c, FPG, Candida colonization density	Hyperglycemia and glucosuria promote fungal growth and tissue adhesion; Candida balanitis is strongly associated with T2DM and IR.

## Data Availability

No new data was generated for this review, but studies were reviewed from publicly available databases.
